# Steroid-induced Lactic Acidosis in Diffuse Large B-cell Lymphoma

**DOI:** 10.7759/cureus.7446

**Published:** 2020-03-28

**Authors:** Daniel Griffin, Rahul Myadam, Parth Patel

**Affiliations:** 1 Pulmonology and Critical Care, University of Missouri-Kansas City, Kansas City, USA; 2 Internal Medicine, University of Missouri-Kansas City, Kansas City, USA; 3 Internal Medicine, University of Missouri-Kansas City School of Medicine, Kansas City, USA

**Keywords:** critical care, tumor lysis syndrome, diffuse large b-cell lymphoma

## Abstract

A previously healthy 53-year-old male with primary membranous nephropathy (positive anti phospholipase A2 antibody) presented to our hospital with worsening cough, shortness of breath, hypotension, and malaise. During his hospital stay, he quickly progressed to overt respiratory failure requiring mechanical ventilation. Upon further workup, he met clinical criteria for tumor lysis syndrome due to an unknown diffuse large B-cell lymphoma, who underwent rapid cytolysis after starting stress dose steroids.

## Introduction

Tumor lysis syndrome (TLS) is an oncologic emergency in which rapid tumor cell lysis leads to the release of intracellular ions into the blood, causing severe electrolyte abnormalities. These electrolyte abnormalities can lead to acute kidney injury, seizures, cardiac arrhythmias, or even death. Large, rapidly proliferating tumors such as Burkitt’s lymphoma, lymphoblastic lymphoma, or acute leukemia are more likely to develop TLS. However, it has been described even with solid tumors such as small cell lung cancer, hepatocellular cancer, and breast cancer [1-3]. Initiation of chemotherapy is the usual trigger for this catastrophe; however, it has been reported even after radiotherapy and immunotherapy [4]. Steroids alone rarely lead to TLS with the first case of steroid-induced TLS described by Dhingra and Newcom in 1988 [5]. We herein present a patient who developed tumor lysis after stress dose steroid use. 

## Case presentation

A 53-year-old male presented to the emergency department with hypotension and a two-week history of shortness of breath, cough, and malaise. He also reported some left-sided chest pain, sweats, and occasional diarrhea. He denied any recent lower extremity edema, nausea, vomiting, fever, abdominal pain, sick contacts. On arrival to the emergency department, he was noted to have a temperature of 97.6 degrees Fahrenheit, a heart rate of 77 beats per minute, a respiratory rate of 26 breaths per minute, a blood pressure of 100/64 mmHg, and an oxygen saturation of 93% on room air. The physical exam of the heart and lungs was unremarkable. The abdominal exam noted an obese abdomen with liver enlargement, and the spleen was not palpable. Laboratory data were notable for a lactic acid of 4.7 mmol/L (reference range 0.0-2.0 mmol/L), a creatinine of 1.5 mg/dL (reference range 0.6-1.3 mg/dL), a blood urea nitrogen of 46 mg/dL (reference range 7-26 mg/dL), and an anion gap of 13 (reference range 5-17). The electrocardiogram noted normal sinus rhythm. A chest x-ray was performed and did not show any acute cardiopulmonary disease. 

On further discussion, the patient had recently been diagnosed with membranous glomerulonephritis and was following with nephrology secondary to this. On the day of admission, he was seen by his nephrologist, who referred him to the emergency department due to a blood pressure of 92/64 mmHg and his reported symptoms of cough, sweats, and increasing shortness of breath. He had been receiving cyclophosphamide and chronic steroids at 60 mg of prednisone daily in alternating cycles for his membranous glomerulonephritis. His last dose of prednisone was approximately one month prior. Due to his current treatment regimen and symptoms, there was increased concern for opportunistic infection, and he was started on broad-spectrum antibiotics and admitted to the intensive care unit. At this time, he was given appropriate intravenous hydration and was started on stress dose hydrocortisone due to his history of chronic prednisone use. After intravenous hydration, his blood pressure had improved to 122/78 mmHg. He remained on room air and stated that his symptoms felt somewhat improved to stable. He was monitored for 24 hours in the intensive care unit. During this time, cultures were negative to date, and his respiratory pathogen panel was positive for rhinovirus. Despite this, his lactic acid continued to remain elevated, ranging from 3.8 to 5.7 mmol/L (reference range 0.0-2.0 mmol/L).

**Figure 1:**
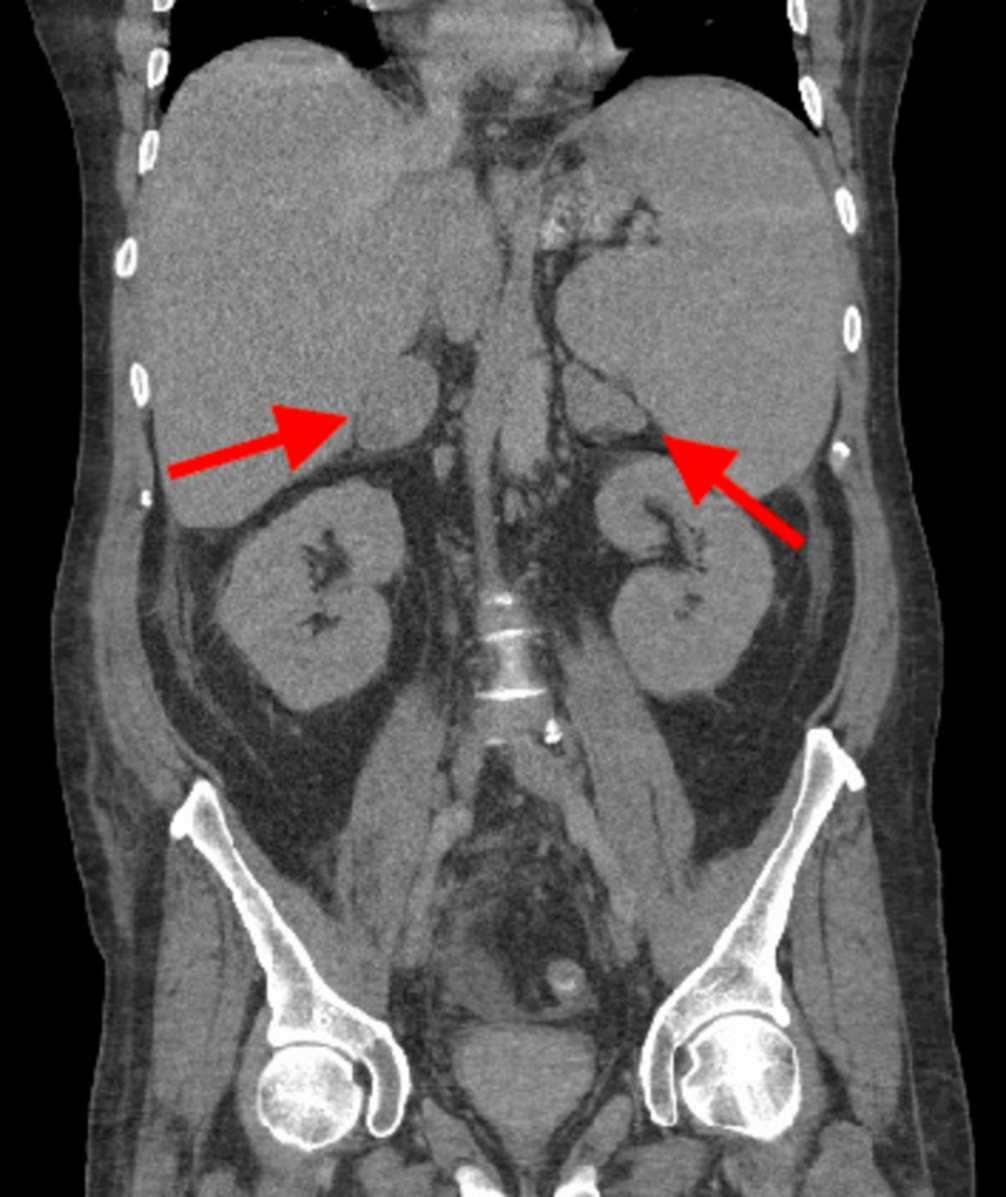
CT of the abdomen and pelvis showing bilateral adrenal masses

Due to this continued lactic acid elevation and reported history of diarrhea, computed tomography of the abdomen and pelvis was ordered. It showed large bilateral adrenal masses and significant abdominal lymphadenopathy but no other acute findings (Figure 1).

**Figure 2:**
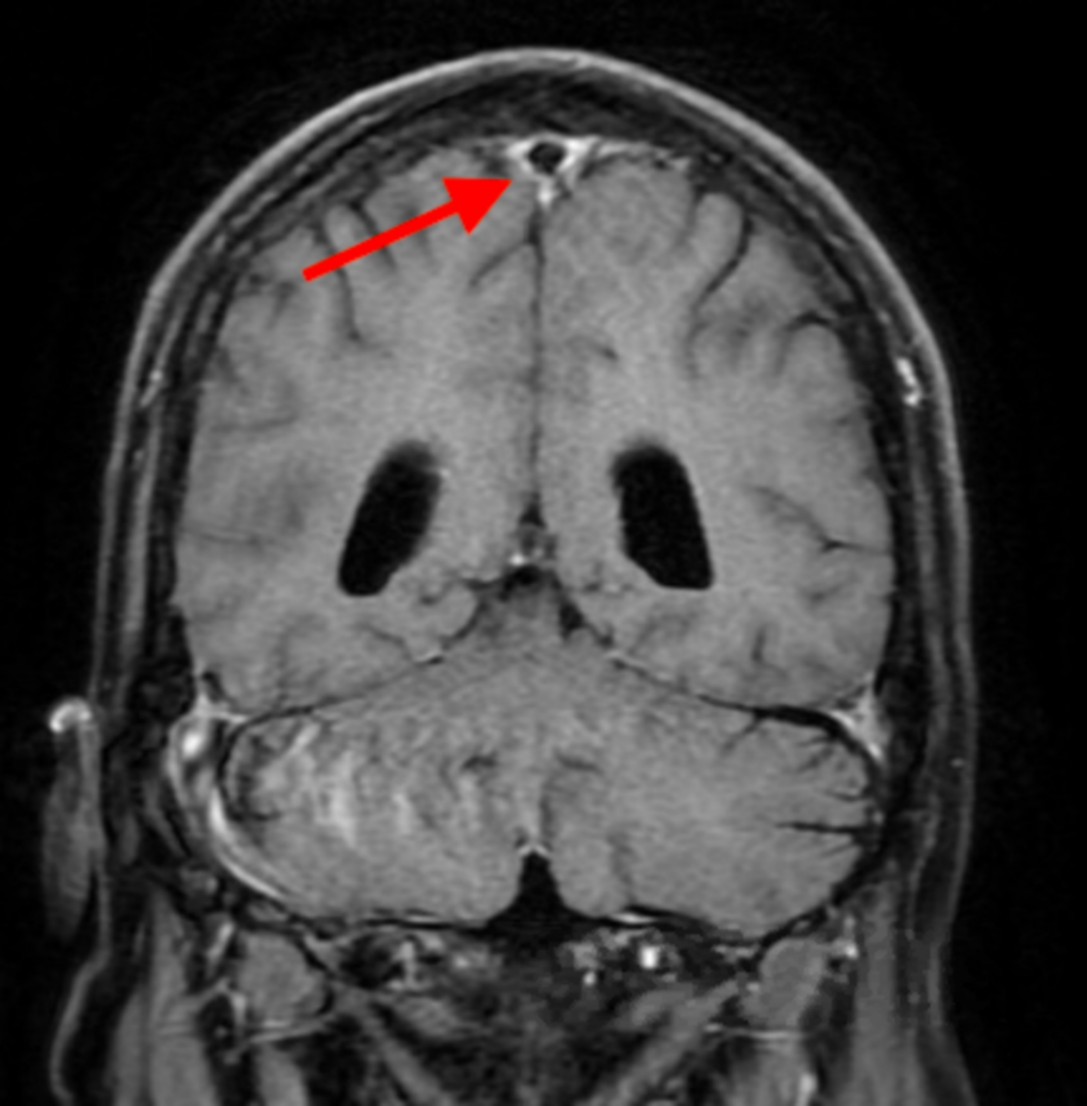
Failure of cerebrospinal fluid suppression within the subarachnoid space

On review of the previous imaging, only an abdominal ultrasound was found which did not bilateral adrenal masses. There was no comment in this study of abdominal lymphadenopathy. Over the next 48 hours, he was noted to become increasingly confused. Cultures remained negative, and he has continued on broad-spectrum antibiotics as well as stress dose steroids. Computed tomography of the head was obtained and did not note any acute findings. Lumbar puncture was performed, which was also negative for an infectious cause of his change in mental status. Magnetic resonance imaging of the head was obtained and noted failure of the cerebrospinal fluid signal to suppress within the subarachnoid space, which can be seen in meningitis, encephalitis, convexity (non-aneurysmal) subarachnoid hemorrhage or leptomeningeal carcinomatosis (Figure 2). 

Over the ensuing days, he continued to have worsening mental status and the development of respiratory failure requiring intubation. Due to his continued acidosis, emergent hemodialysis was initiated (Table 1). Follow-up laboratory data showed a uric acid of 21.1 mg/dL (reference range 2.5-7.0 mg/dL) and a lactate dehydrogenase level of greater than 25,800 IU/L (reference range 313-618 IU/L). At this point, there was a significant concern for TLS, and a bone marrow biopsy was obtained, which showed diffuse large B-cell lymphoma (DLBCL) and confirmed TLS. He was continued on treatment for his TLS and was started on chemotherapy. Over the following days, he was able to be extubated, and his mental status returned to normal. He was continued on hemodialysis and chemotherapy while in the hospital over the next several weeks, but was subsequently able to be discharged home without the need for long-term dialysis. 

**Table 1:** Lactic acid levels Lactic acid levels from arrival to resolution.

Lactic acid (reference range 0.0-2.0 mmol/L)	Clinical data
4.7	Initial value on arrival. Intravenous fluids (IVF), antibiotics, and steroids started.
2.5	Repeat value after aggressive IVF.
2.9	Over the next two days, lactic acid levels remain elevated despite aggressive IVF. Additional IVF stopped during this time due to concerns for volume overload.
3.2	
3.8	
4.1	
3.7	
4.1	
3.9	
4.1	Lactic acid levels continue to trend up.
4.6	
5.3	
6.8	
7.7	
8.4	
9.3	
10.8	
11.5	Dialysis initiated.
8.9 – 1.7	Lactic acid levels normalize after dialysis and chemotherapy.
1.3	Lactic acid levels remain normal at the time of discharge off dialysis.

On discussion with oncology, it was felt that the patient membranous glomerulonephritis was secondary to his DLBCL and that he went into TLS secondary to the stress dose steroids, which were started on admission. 

## Discussion

DLBCL is the most common type of non-Hodgkins lymphoma and accounts for 25% of cases [6]. Patients typically present with “B” symptoms (fever, weight loss, night sweats) and a rapidly growing mass. The diagnosis of DLBCL is often made based on excisional tissue biopsy [7]. It can be challenging to recognize a patient presenting with such malignancy due to its ability to mimic other disease processes such as sepsis. 

A typical laboratory abnormality that is seen in many disease processes is lactic acidosis [8]. Lactate is a by-product of anaerobic metabolism. Lactic acidosis is one of the most common causes of metabolic acidosis in patients admitted to the hospital, and patients with advanced malignancies commonly present with type B lactic acidosis [9]. Systemic hypoperfusion is not apparent in this type of lactic acidosis, unlike type A lactic acidosis, which can be seen with bacteremia [10]. This type of lactic acidosis often goes unrecognized and mistreated. Multiple theories have been proposed; however, the pathophysiology of this occurrence in advanced malignancies is still not well understood [11-13]. Regardless of the mechanism, treatment of the malignancy will usually correct the lactic acidosis as it was seen with our patient. Physicians should have malignancy as a differential with a lactic acidosis that does not correct despite adequate fluid resuscitation. 

While TLS is a common feature in malignancy, it is a medical emergency that requires prompt initiation of treatment and prevention. TLS is most commonly related to lymphomas and leukemias. A majority of the TLS cases that have been reported occur after chemotherapy. Less commonly, TLS has been caused by glucocorticoid therapy, as with the patient presented [14,15]. Physicians should use glucocorticoids in patients with a diagnosis of malignancy cautiously, and when malignancy is high on the differential. 

## Conclusions

Malignancy should be considered in the differential for a lactic acidosis without apparent systemic hypoperfusion. Glucocorticoids should be cautiously used in patients with untreated malignancy or when malignancy is high on the differential.
